# Synergistic Effect of Retinoic Acid and Lactoferrin in the Maintenance of Gut Homeostasis

**DOI:** 10.3390/biom14010078

**Published:** 2024-01-08

**Authors:** Ma. Concepción Peña-Juárez, Omar Rodrigo Guadarrama-Escobar, Pablo Serrano-Castañeda, Abraham Méndez-Albores, Alma Vázquez-Durán, Ricardo Vera-Graziano, Betsabé Rodríguez-Pérez, Mariana Salgado-Machuca, Ericka Anguiano-Almazán, Miriam Isabel Morales-Florido, Isabel Marlene Rodríguez-Cruz, José Juan Escobar-Chávez

**Affiliations:** 1 Unidad de Investigación Multidisciplinaria Lab-12 (Sistemas Transdérmicos y Materiales Nanoestructurados), Facultad de Estudios Superiores Cuautitlán, Universidad Nacional Autónoma de México, Carretera Cuautitlán Teoloyucan, Km 2.5, San Sebastián Xhala, Cuautitlán Izcalli 54714, Mexico; maconcepcionpenajuarez@gmail.com (M.C.P.-J.); escobaromarrodrigo@gmail.com (O.R.G.-E.); pabloqfb@hotmail.com (P.S.-C.); marianasalgado9401@gmail.com (M.S.-M.); eri.qa.30@hotmail.com (E.A.-A.); mflorido.cf@gmail.com (M.I.M.-F.); 2 Unidad de Investigación Multidisciplinaria Lab-14 (Ciencia y Tecnología de los Materiales), Facultad de Estudios Superiores Cuautitlán, Universidad Nacional Autónoma de México, Carretera Cuautitlán Teoloyucan, Km 2.5, San Sebastián Xhala, Cuautitlán Izcalli 54714, Mexico; albores@unam.mx (A.M.-A.); almavazquez@comunidad.unam.mx (A.V.-D.); 3 Instituto de Investigaciones en Materiales, Universidad Nacional Autónoma de México, Apartado Postal 70-360, Ciudad Universitaria, Coyoacán, Ciudad de México 04510, Mexico; graziano@unam.mx; 4 Laboratorio de Servicio de Análisis de Propóleos (LASAP), Unidad de Investigación Multidisciplinaria (UIM), Facultad de Estudios Superiores Cuautitlán, Universidad Nacional Autónoma de México, Cuautitlán Izcalli 54714, Mexico; berope380@hotmail.com; 5 Laboratorio de Farmacia Molecular y Liberación Controlada, Departamento de Sistemas Biológicos, Universidad Autónoma Metropolitana, Mexico City 04960, Mexico; 6 Unidad de Enseñanza e Investigación, Hospital Regional e Alta Especialidad de Sumpango, Carretera Zumpango-Jilotzingo #400, Barrio de Santiago, 2ª Sección, Zumpango 55600, Mexico; isabelmarlen05@gmail.com

**Keywords:** lactoferrin, intestinal microbiota, retinoic acid, gut immunity, immunomodulation, dysbacteriosis, eubiotic state

## Abstract

Lactoferrin (LF) is a glycoprotein that binds to iron ions (Fe^2+^) and other metallic ions, such as Mg^2+^, Zn^2+^, and Cu^2+^, and has antibacterial and immunomodulatory properties. The antibacterial properties of LF are due to its ability to sequester iron. The immunomodulatory capability of LF promotes homeostasis in the enteric environment, acting directly on the beneficial microbiota. LF can modulate antigen-presenting cell (APC) biology, including migration and cell activation. Nonetheless, some gut microbiota strains produce toxic metabolites, and APCs are responsible for initiating the process that inhibits the inflammatory response against them. Thus, eliminating harmful strains lowers the risk of inducing chronic inflammation, and consequently, metabolic disease, which can progress to type 2 diabetes mellitus (T2DM). LF and retinoic acid (RA) exhibit immunomodulatory properties such as decreasing cytokine production, thus modifying the inflammatory response. Their activities have been observed both in vitro and in vivo. The combined, simultaneous effect of these molecules has not been studied; however, the synergistic effect of LF and RA may be employed for enhancing the secretion of humoral factors, such as IgA. We speculate that the combination of LF and RA could be a potential prophylactic alternative for the treatment of metabolic dysregulations such as T2DM. The present review focuses on the importance of a healthy diet for a balanced gut and describes how probiotics and prebiotics with immunomodulatory activity as well as inductors of differentiation and cell proliferation could be acquired directly from the diet or indirectly through the oral administration of formulations aimed to maintain gut health or restore a eubiotic state in an intestinal environment that has been dysregulated by external factors such as stress and a high-fat diet.

## 1. Introduction

In an individual with a healthy and stable gut microbiota, there are approximately 10^14^ microorganisms living symbiotically with the human host. Their functions include maintaining health via vitamin synthesis, fermenting dietary fibers, xenobiotic metabolism, and pathogen defense [1], which are necessary for the maintenance of homeostasis [2].

Metabolic disorder or metabolic syndrome (MetS) is a set of organic disorders that occur at the same time and in scenarios that promote their prevalence and contribute to chronic disease development. Being overweight and obese are the main determinants (i.e., risk factors) for MetS development [3]. MetS is a condition associated with a gut microbiota imbalance. Progression to any metabolic disorder is mainly associated with genetic predisposition but also with an inappropriate diet, such as a high-fat diet (HFD), which promotes oxidative stress. Oxidative stress is generated by the imbalance between reactive oxygen species (ROS) and antioxidant enzymes in tissues such as the liver and can also be influenced by gut inflammation [4,5].

An imbalanced gut microbiota (known as dysbacteriosis) is related to high carbohydrate consumption [6] and is associated with insulin resistance. Both fats and carbohydrates trigger important downregulated processes and chronic inflammation, which are determinants of the onset of chronic disease.

The state in which the microbiota is healthy and balanced is known as the eubiotic state and includes a mutualistic association between numerous bacterial species. The eubiotic state is the condition in which the host fully benefits from the metabolites and products of the microbiota [7].

Both the eubiotic state and dysbacteriosis are influenced by the environment, genotype, and diet, and both influence health and disease in the host because of the development of an inflammatory state that can be reversed when the causative effect disappears [8].

When the intestinal environment has been damaged, the microbiota or their metabolic products can enter the circulation and cause inflammation and damage. The initial stage of chronic inflammation can be reversed with the help of anti-inflammatory agents [9].

A means to counteract the damage caused by chronic inflammation is the oral administration of probiotic and prebiotic compounds, which can improve the function of the microbiota and hamper the production of highly toxic metabolites in the host. The regular consumption of probiotics promotes the maintenance of a healthy intestinal immune system as well as the maturation and communication of intestinal cells with the various microbiota populations because probiotics act on regulatory T-cells (Tregs) [10]. Similarly, the consumption of prebiotics stimulates the growth of healthy microbiota populations. However, if an imbalance has been initiated and metabolic disorders have already been triggered, the microbiota recovery process will be slower, conventional probiotics alone will not be able to balance the microbiota, and prebiotics will lose their effectiveness on the new populations of probiotics that have been introduced to the intestinal environment [11].

Certain natural molecules derived from the diet, such as vitamins [12], probiotics, and biologics [13], have demonstrated interesting properties regarding the correction of problems related to chronic gut inflammatory processes and microbiota imbalances. Two such molecules with probiotic activity are human and bovine lactoferrin (LF) and retinoic acid (RA).

LF, a glycoprotein with a high affinity for iron, is a member of the transferrin family and has antimicrobial properties [14]. LF maintains iron homeostasis, promotes immune regulation through anti-inflammatory and antioxidant activity, and has antibacterial and antitumor effects. Thus, LF can help prevent the development of diseases and can promote human health in general [15].

X-ray crystallographic analysis has revealed the three-dimensional configuration of LF, unveiling a globular protein with two remarkably homologous lobes able to bind a ferric ion each [16]. LF expression begins during embryonic development at the blastocyst stage. After a period of latency, its expression resumes until the second half of gestation, being observed in neutrophils and epithelial cells of the digestive and respiratory tract. On the other hand, in the adult stage, LF is secreted in the mucosal fluids and glandular epithelial cells, but its most elevated concentrations are found in colostrum and milk, while comparatively reduced levels are observed in tears, nasal fluids, saliva, and secretions from pancreatic, gastrointestinal, and reproductive tissues [17].

The high concentration of LF and iron bioavailability in breast milk suggest that LF may play a critical role in intestinal iron absorption in the neonate [17].

Retinol, retinal, and retinyl esters are members of the vitamin A group, which encompasses these fat-soluble retinoids. Humans are incapable of synthesizing vitamin A (VA), so its uptake from the diet is essential. Vitamin A is acquired either in this form from animal products or as provitamin carotenoids (e.g., beta-carotene) from plants [18]. Retinol is a precursor and a transport form and is activated to retinoic acid (RA) by two enzymatic reactions [19].

In mice, where the transformation process has been mainly analyzed, the first reaction produces retinaldehyde via retinol dehydrogenase-10 (RDH10) activity. The second reaction is the conversion of retinaldehyde to RA by the action of three retinaldehyde dehydrogenases: ALDH1A1, ALDH1A2, and ALDH1A3 [20].

Vitamin A plays a crucial role in numerous physiological functions, including vision, growth, reproduction, hematopoiesis, and immunity. It stands as one of the most extensively studied nutrients concerning immune function. The initial observations indicating a connection between vitamin A and immunity predate the elucidation of vitamin A’s structure in 1931. These early findings included the recognition that the fat in butter enhanced infection outcomes in malnourished animals and the observation that rats deficient in vitamin A seemed more vulnerable to infections [18].

Both LF and RA are present in high quantities in colostrum, human milk, and mucosa. They are also related to the activation and regulation of the immune system and have been reported to possess synergic effects in this regard, improving class-switching to IgA and stimulating its production. Synergistically, LF and RA can increase the receptor expression for homing molecules like CCR9 and α4β7 in B-lymphocytes (LBs) (Table 1). These characteristics are observed in the intestinal lamina propria (LP) [21]. Similarly, RA and microbial products like short-chain fatty acids (SCFAs) encourage the development and differentiation of Treg lymphocytes [22].

In this review, we highlight the most important functions of LF and RA in the regulation of the gut microbiota balance to reduce inflammation and prevent the development of insulin resistance, metabolic disorders, and their progression to obesity and diabetes. We propose the synergistic use of both molecules in the prophylactic treatment of metabolic disorders to avoid the development of diabetes.

## 2. The Intestinal Environment in Health

### 2.1. Antigen-Presenting Cells

Antigen-presenting cells (APCs), monocytes/macrophages (Mos/MØs), dendritic cells (DCs), and LBs are responsible for generating an effective immune response against pathogens. If the microorganisms persist, these cells can display peptides processed via major histocompatibility complexes I or II (MHCI or MHCII) to TCD4 or TCD8 lymphocytes. When these cells are activated, they secrete cytokines such as IL-2, which promote lymphocyte proliferation and differentiation [23]. Subsequently, they secrete interferon-gamma (IFN-γ), which enables them to exert the effector functions of MØ for the elimination of pathogens. In the intestine, pathogenic microorganisms are captured by DCs or specialized epithelial cells called M-cells, which can capture them intact and transport them along with molecules from the intestinal lumen through the mucosal barrier, from the apical part of the dome located above the gut-associated lymphoid tissue (GALT) to make them available to DCs in the underlying lymphoid tissue [24]. Following antigen capture, the DCs are activated and express co-stimulatory B7 molecules (1 and 2) that allow them to present the antigen to TCD4 cells, which are subsequently differentiated into memory or effector cells [20]. The latter activates MØs to phagocytize and LBs to facilitate the removal of pathogens by secreting specific antibodies (Abs) [25].

Furthermore, intestinal antigen-presenting cells (IAPCs) help maintain tissue homeostasis from PRRs in the microbial cell membranes. These IAPCs detect commensal bacteria and contribute to the resistance to tissue injury, which may be triggered by these PRRs, initiating the inflammatory pathway signaling. Resistance to tissue injury pathways may include caspase-1, nuclear factor NFkB, and mitogen-activated protein kinase (MAPK) production. This plays a role not only in stopping infection and bacterial colonization but also in regulating the intestinal barrier function, epithelial repair, and homeostasis [26,27].

Furthermore, these APCs (DCs specifically) contribute by generating TH17 cells, which secrete proinflammatory cytokines such as IL-22 in response to the modulation of the intestinal immune system, followed by microbiota changes that may be responsible for some degree of inflammation [28]. Therefore, these APCs are responsible for maintaining tolerance to food antigens and intestinal microbiota without diminishing their ability to react against pathogens that endanger the integrity of the epithelial barrier [29].

Moreover, in the intestine, DCs are the only cells able to convert vitamin A to RA [30]. RA is essential for the regulation of immune functions, such as the homing of lymphocyte populations, and for reducing the risk of infection. RA is required for normal function regarding visual capacity, cell growth, maintenance of epithelial integrity, and the production of blood cells. In addition, RA is a metabolite that induces tropism and the intestinal functional differentiation of T-cells and both the LP and stromal cells (SCs). When vitamin A is incorporated into the mucosal epithelium, its products are quickly incorporated into the tissue to carry out regulatory activity toward cell differentiation and growth of the intestinal barrier [31]. Some investigations have reported the participation of an LP intestinal SC population that can induce the production of RA by DCs in an RA-granulocyte-macrophage colony-stimulating factor (GM-CSF)-dependent fashion [32].

### 2.2. Dendritic Cells and Macrophages

DCs are a heterogeneous population of highly specialized cells and the most important components of the immune system [33]. They are also the most effective APCs, inducing a response due to their outstanding capacity to activate naive TCD4 lymphocytes, which are the bridge between innate and adaptive immunity. DCs are also capable of controlling immunity, as is the case of CD103+ resident cells in the mesenteric lymph node (MsnLN) and LP (which secrete RA) and transforming growth factor-beta (TGF-β), thereby promoting the generation of regulatory Foxp3+ Treg cells and contributing to the differentiation of IgA-secreting plasma cells [34]. The latter cells are specialized in communication with T-cells via antigen presentation to naïve TCD4 cells, activating them and triggering an adaptive response, inducing tolerance [35]. Nevertheless, when resident DCs present the antigen in the absence of co-stimulation, it might not trigger an immune response, in turn slowing down the reactivity of the T-cells [36].

Mos/MØs are phagocytic immune cells that connect the innate and adaptive response through the antigenic processing of extracellular pathogens and their presentation via MHCII. Mos/MØs are part of the first line of host defense and the primary phagocytes performing processing and elimination by internalizing foreign particles in lysosomes [37]. These phagocytes can detect pathogens and parasitic organisms via pattern recognition receptors (PRRs) and the toll-like receptor (TLR), which can activate several receptors at once [38]. Mos/MØs secrete proinflammatory cytokines such as IL-12, TNF-α, and monocyte chemoattractant protein 1 (MCP-1), which increase the innate immune response, and IL-10, which regulates the immune response. Mos/MØs may also activate the adaptive immune response after antigen presentation to TCD4 cells by recognizing MHCII receptors. On the other hand, they secrete cytokines to establish homeostasis in the immune cellular network and participate in the maintenance and re-stimulation of T-lymphocytes contained in the LP. Mos/MØs express molecules such as CD64, the immunoglobulin Fc receptor (FcꝩRI), CX3CR1 chemokine receptor, F4/80 antigen, epidermal growth factors (EGFs) such as the mucin-like hormone-containing module (1-EMR1), CD11b, and CD11c [39].

Moreover, MØs are involved in maintaining tissue homeostasis by expressing the interleukin-10 receptor (IL-10R) on their surface. The corresponding cytokine, when bound to its receptor on MØs, suppresses those associated with inflammation, upholding a non-inflammatory status and contributing to the maintenance of homeostasis. Equally importantly, MØs are involved in the tissue repair process and type II inflammatory response [40,41].

### 2.3. The Intestinal Microbiota

The intestinal lumen is inhabited by about 100 trillion commensal bacteria [42] consisting of around 2000 species, dominated by anaerobic bacteria, yeasts, and viruses—predominantly bacteriophages that are responsible for lysing bacteria, and have a great influence on strain growth modulation and maintaining the diversity of microbial species [43]. The homeostasis between the host and the microbiota is essential for the maintenance of the eubiotic state, which is critical for health, as intestinal homeostasis prevents the development of inflammatory reactions, which can trigger processes of hyper-responsiveness to food components [44]. In the intestinal environment, there are three bacterial phyla, namely Bacteroidetes, Firmicutes, and Actinobacteria, among which the Bacteroidetes phylum comprises Gram-negative bacteria. The main factors that maintain the balance between the gut microbiota and gut secretions are gastric acid, mucus, biliary salts, immunoglobulins, the mucosal barrier, intestinal motility, the immunological system, and interactions between the different bacterial strains. This balance plays an essential role in energy collection and the capture and storage of minerals and bioactive compounds that fulfill functions in the body that can promote good health. If any components of this balance are affected, chronic inflammatory processes may develop, such as those in inflammatory bowel disease, Crohn’s disease, or ulcerative colitis [45]. On the other hand, a mechanism that promotes cell proliferation in the liver and that depends on the presence of Gram-negative bacteria (Bacteroidetes), occurring in response to the presence of lipopolysaccharide (LPS) has been observed. This mechanism can induce DNA synthesis. This process was noticed in mice in whose livers LPS was removed [46]. In this case, the presence of Gram-positive bacteria (Firmicutes) was regulated by Gram-negative bacteria (Bacteroidetes).

Probiotics are microorganisms able to restore the intestinal microbiota and can be strengthened by prebiotics, which are food ingredients with modulating activity on the intestinal microbiota [47,48].

The main beneficial probiotic populations are the Firmicutes and Bacteroidetes groups (which include the lactobacilli and bifidobacteria, respectively) [49,50]. LF influences these populations as a prebiotic compound; its effects on bifidobacteria have been better studied, and the presence of bLF-binding proteins both in the membrane and in the cytosol has been found to have a bifidogenic effect [51,52].

## 3. Lactoferrin

LF is an 80 kD glycoprotein that is found abundantly in biological materials secreted by mammals. LF has a great affinity for iron, binding two iron ions per molecule. Its main function is to transport iron through the bloodstream to storage sites, such as the liver or bone marrow, where red blood cells are produced [53]. This glycoprotein can be found in its iron-free form, known as apo-lactoferrin, in many mucosal epithelial cells released in the inflammatory process by neutrophils. LF is essential for the immune system of mammals because it is produced as a defense against pathogens such as bacteria, viruses, fungi, and parasites [54]. LF is especially important as a first host response against exogenous infections and endogenous signal injuries. LF has been included in the alarmin group of endogenous mediators thanks to its ability to recruit and activate inflammatory cells [55,56], including APCs that initiate innate and adaptive immune responses [57]. In the intestine, LF can regulate the quantity of iron absorbed through its role in iron transport or iron chelation in a direct or indirect form. Interestingly, LF has been employed in adjuvant therapies for some intestinal diseases, as well as in food products such as nutraceuticals and infant formula [58].

Some LF peptide derivatives [59], as well as recombinant versions of bovine LF [60] and synthetic peptides [61], have been studied due to their promising pharmaceutical applications in the treatment of many microbial diseases caused by pathogenic microorganisms and as immunity mediators.

LF has been associated with various biological processes including host defense, growth, differentiation, and cell function regulation [62]. Specific receptors for LF in blood cells [63] and hepatocytes [64] have also been observed.

In vitro studies in which LF can induce myeloid-derived suppressor cells with high gene expression related to antimicrobial activity and a higher ability to eliminate pathogens have been reported [65]. In an in vivo infection model, lactoferricin B (LFcin B) was able to modulate the abnormal growth of a Bacteroides population, caused by an enterohemorrhagic *E. coli* (EHEC O157:H7) strain, accompanied by a local intestinal inflammatory response. Moreover, serum LFcin B (an antimicrobial lactoferrin-derived peptide) was able to modulate the inflammatory process caused by the *E. coli* infection [66]. In vitro and in vivo studies showed that human LF (hLF) was able to suppress tumor necrosis factor-alpha (TNF-α), interleukin (IL)-6, and IL-1 expression by mononuclear cells in response to lipopolysaccharides (LPS) and could regulate inflammatory processes [67], such as colitis amelioration in rats, by enhancing IL-4 and IL-10 cytokine production [68].

LF can mediate such functions due to its high activity on components of immunity, including humoral and cellular factors. LF has been studied for potential therapeutic applications based on its multifunctional properties, including antimicrobial, antiviral, antifungal, anti-parasitic, anticarcinogenic, and regenerative properties [69] associated with its iron-sequestering capability. Thus, LF shows effectiveness in the treatment of some diseases such as metabolic dysfunctions [70] and osteoporosis [71,72]. LF has also been studied for its nutritional value [73,74].

LF can regulate the immune response because it has antitumor, antimicrobial, and anti-inflammatory activities, and can also modulate the innate and adaptive immune response [75]. The high antioxidant, immunomodulatory, and anti-inflammatory activities of LF occur through signaling pathways, including those of some TLRs [38].

The physiological balance of the ROS production and elimination rate through iron sequestration can be controlled by LF, which has regulatory activity on cellular redox by upregulation of antioxidant enzymes [76].

Through its capability of Fe^3+^ sequestration, LF can prevent the harmful effects of oxidative stress.

Furthermore, LF can hamper the reactivity of free ferric ions with superoxide molecules. This, in turn, reduces the ground-state oxygen and hinders ferrous salt formation, preventing the Fenton reaction (i.e., where there is a hydroxyl radical and hydroxide ion formation and the ferrous ion is oxidized by hydrogen peroxide to ferric ion) [77].

Another function of LF is the homeostasis of iron to protect against oxidative stress, thus preventing cell damage. There are pathophysiologic conditions associated with disruptions of iron homeostasis, including anemia and disorders related to Fe overload [78], because LF is a key component in the iron absorption process as well as the transport and delivery of Fe into the cells.

The antitumor activity of LF has been studied in colon cancer cells, where LF has been shown to affect cell proliferation and invasion in nude mice bearing HT29 tumors [79].

The LFs most investigated for their functionality include LFs of bovine and human origin; in both animal species, LF has remarkably similar characteristics in terms of its amino acid sequence and function [80].

**Figure 1:**
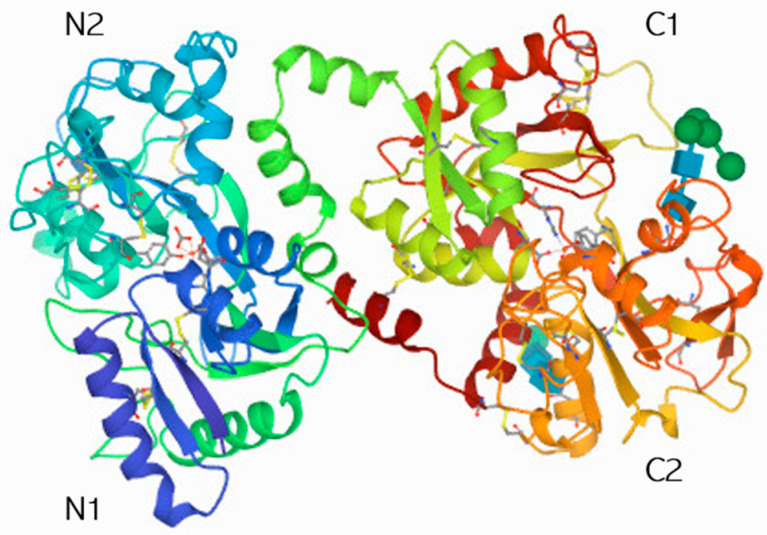
Crystal structure of lactoferrin. Protein data http://www.rcsb.org/pdb/explore.do?StructureId=1BLF (accessed on 26 September 2020).

As shown in Figure 1, two symmetric globular lobes (the N-lobe and C-lobe) are observed, joined by a short α-helix. The lobes are divided into two sub-domains, N1 and N2 and C1 and C2, which are similarly sized [81]. Moreover, the surface of the protein is positively charged, which allows its binding to anionic compounds.

### 3.1. Effect of Lactoferrin on Dendritic Cells and Macrophages

In addition to immune cells, many cell types can bind LF. However, the binding is performed according to the species, cell, and type of tissue [82]. Furthermore, studies have reported the direct binding of LF to Mos/MØs and DCs employing surface receptors such as C-X-C-motif cytokine receptor 4 (CXCR4), receptor-related protein-1 (LRP-1/CD91), intelectin-1 (omentin-1), and TLR4 [83].

The first mammalian cell type that was reported to bind to LF was mouse peritoneal MØs, with the presence of a specific receptor for this purpose, the lactoferrin receptor (LFR) [84,85]. The main effects of LF on DCs were studied in DCs derived from macrophages (MC-DCs). As a result, knowledge about differentiation effects in these immune cell lines was gained.

**Figure 2:**
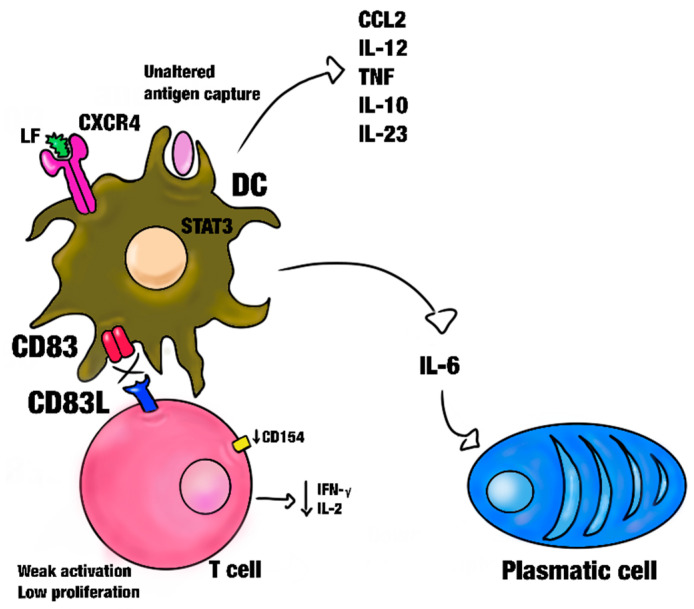
CD83 is a DC maturation marker and it is expressed in MC-DC thymus, circulating DCs, DCs Langerhans, the spleen interdigitating reticulum cells, and thymic medulla DCs. These are required for an effective activation of naive TCD4 cells. LF promotes the differentiation of DC without CD83/CD83L participation, so these DCs maintain a state of immaturity. Thus, there is a weak activation and low proliferation in T-cells, and a decrease in the CD154 marker as well as altered expression of IFNγ and IL-2. The LF-DC activation promotes IL-6 secretion.

In the presence of LF, the differentiation and function of undifferentiated MC-DCs are promoted. These MC-DC-derived cells experience overexpression of the CD83 surface marker but are not activated in this way [86]. CD83, which is an indicator of DC maturation and essential for naïve TCD4 cell activation at the time of antigen presentation and the subsequent generation of Tregs, fails to undergo activation like MHC and co-stimulatory complex molecules, as well as cytokine and chemokine secretion. CD83-deficient DCs produce high levels of IL-12 and boost the expression of CD25 and OX40L co-stimulatory molecules, promoting a higher antigen-specific T-cell response and affecting the Treg population and its suppressive functions [87]. A decrease in or the inhibition of the cell surface expression of CD83 has as a consequence the inability to stimulate T-cells (Figure 2). This causes a decreased secretion of IFN-γ by T-cells and, similarly, the ability to attract the TCD8+ cell tumor-specific antigen [88]. Moreover, LF suppresses MHC expression and co-stimulatory molecules in DCs and interferes with the secretion of some cytokines in T-cells, such as INF-γ and IL-2. However, MC-DCs differentiated in the presence of LF exhibit increased expression of molecules such as the immunoglobulin-like transcript and programmed death ligand (indoleamine 2,3-dioxygenase and suppressor of cytokine signaling 3), which have negative regulatory or inhibitory immune functions [89].

Interestingly, these MC-DCs express IL-6 [54], which is involved in the differentiation of B-cells and the activation of plasmatic and cytotoxic T-lymphocytes, modulation of the growth and differentiation of immune cells [90], and can influence brown adipose tissue (BAT) metabolism. IL-6 also stimulates the secretion of cytokines such as CCL2, IL-12, TNF, IL-10, and IL-23, which activate DCs [79].

LF has been reported to selectively activate MØ/TRL-4-dependent signal pathways and induce them to express CD40 and secrete IL-6 [91]. In an infectious vesicular stomatitis virus (VSV) model, peritoneal macrophages (PMs) exposed to a viral infection could form LF/LPS complexes and initiate a TLR-4-dependent signal pathway, inducing IFN-α/β production and viral infection remission, upon the addition of LF [92]. Furthermore, LF can enhance the NK response in a dose-dependent manner, but in the case of antibody-dependent cellular cytotoxicity (ADCC), the opposite effect is observed, wherein LF has an inhibitory effect at the same concentration [93,94]. In BCG-infected murine bone marrow-derived macrophages (BMMQs) exposed to bovine lactoferrin (bLF), this can regulate the expression of MHCI and II [95]. bLF has also been reported to modulate the basal production of LPS-induced cytokines in naive Mos/MØs, such as IFN-ß, IL-12, IL-6, IL-8, and TNF-α [82,91,96]. In activated MØs, IL-8 secretion is inhibited by contact with bLF [97].

There is also evidence that CXCR4 could act as a receptor for LF and mediate the PI-3K/Akt signaling pathway [83], which modulates cellular functions.

### 3.2. Effect of Lactoferrin on the Intestinal Microbiota

At birth, humans require adequate nutrition to develop a healthy intestinal bacterial environment (i.e., the intestinal microbiota), which allows for the development of an appropriate intestinal immune system and immune gut homeostasis [98]. This process occurs mainly through breastfeeding and the action of its components such as LF and lysozyme. Although individuals at birth are fully capable of mounting an immune response, immune effector components require bacterial challenge. This is achieved by drinking maternal milk, whose components stimulate the proliferation of the microbiota, which populate the intestinal epithelium in a balanced and diverse way. The microbiota is responsible for transforming the intestinal TH2 response in the sterile intrauterine environment to Th1/Th2 activation. T-cell activation is necessary for this and can be achieved by specific agents present during breastfeeding, such as bifidobacteria, lactobacilli, and Bacteroides [99]. The main effects of LF on immune gut cells are listed in Table 2.

A complex process underlies lactoferrin’s advantageous effects on bifidobacteria, supporting the development and activity of these good bacteria in the digestive tract. A glycoprotein found in milk called lactoferrin functions as a prebiotic by favorably promoting the growth of bifidobacteria [108]. One important mechanism is lactoferrin’s capacity to bind to iron, which limits the amount of iron available to harmful bacteria and fosters the growth of bifidobacteria, which are well-suited to environments with reduced iron levels [108]. Furthermore, lactoferrin has antibacterial qualities that prevent the formation of unwanted bacteria, giving bifidobacteria a competitive edge. Additionally, this glycoprotein has immuno-modulatory properties that affect the host’s immunological response and foster an environment that is favorable for bifidobacteria to thrive in. Furthermore, lactoferrin helps to improve the function of the gut barrier, which supports the colonization and survival of bifidobacteria in the intestinal mucosa [109]. Lactoferrin’s bifidogenic effects could be defined by a variety of complex mechanisms that work in concert to promote a symbiotic interaction between bifidobacteria and this glycoprotein, ultimately aiding in the development of a balanced and healthy gut microbiota.

In newborns, healthy microbiota such as Bacteroides can bind IgA, which allows them to colonize the gastrointestinal tract, promoting immune system development and microbiota colonization [110].

LF and lysozyme actively participate in the composition of healthy microbiota, mainly comprising lactobacilli and bifidobacteria [111]. LF acts as a growth promoter [100], which is the main immune factor involved in host defense against pathogens, in addition to acting as an anti-inflammatory agent [112,113].

**Figure 3:**
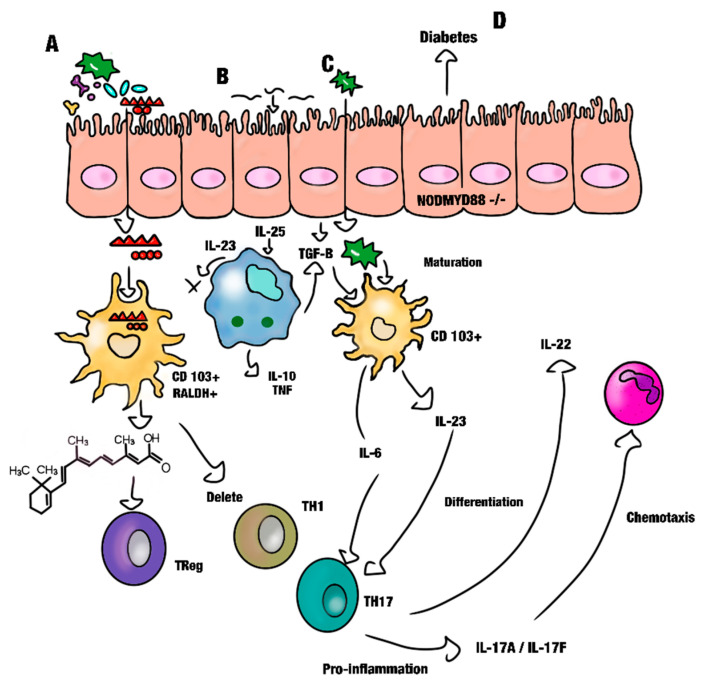
(**A**) Retinoic acid (RA) can promote the bifidobacterial growth, which secrete polysaccharides that induce DC to secrete the retinoic acid and subsequently induces Treg differentiation. Immunomodulation by the bifidobacteria is a mechanism that can be both dependent and independent of RA. (**B**) Enterocytes secrete TGFβ-promoting maturation DC to secrete RA and in turn the latter will favor the differentiation of Treg. (**C**) The TGF-β and LF induce differentiation of TH17 that secretes IL-22, maintaining intestinal homeostasis and stimulates secretion of antimicrobial peptides. (**D**) Diabetes develops in free microbiota environmental in mice.

The influence of the microbiota on DC populations has been analyzed in some reports [101]. It is well known that DCs can induce TCD4^+^ cell differentiation, and these T-cells can secrete IL-17 and IL-22. When the epithelium is colonized by segmented filamentous bacteria, the expression of genes associated with inflammation and antimicrobial properties increases, thus augmenting the risk of developing diabetes or autoimmune diseases (Figure 3) [102]. This mechanism has not been fully described yet. It has been observed that mice unresponsive to TLR signals or those with Myd88 deficiency do not develop diabetes. Moreover, knockout NOD/Myd88-/-mice in germ-free conditions develop diabetes, which can be countered when defined microbiota communities are transferred [103]. This supports the idea that maintaining the homeostasis between immunity through DCs and the microbiota is critical to prevent dysbiosis and the subsequent development of metabolic disorders and clinical signs such as diabetes or inflammatory bowel disease [104].

LF has saturation levels that depend on three of its forms: iron-depleted LF (apoLF), monoferric LF, and iron-saturated LF (holoLF). Both native and iron-saturated LF can restore normal levels of anti-inflammatory bacteria after clindamycin administration, which induces intestinal microbiota alterations such as a decrease in the population of anti-inflammatory bacteria (Prevotellaceae or Rikenellaceae and Bacteroidaceae), in addition to restoring normal levels of TLR2, TLR8, and TLR9 receptor expression. However, TLR receptor expression can be restored only by iron-saturated LF [107].

## 4. Retinoic Acid (RA)

**Figure 4:**
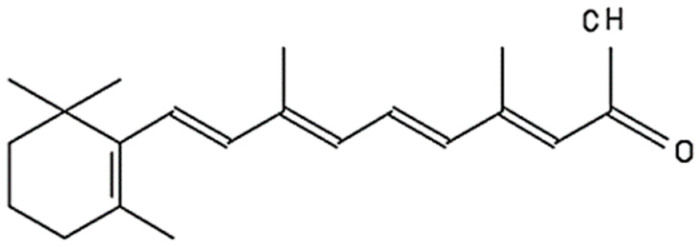
All-trans-Retinoic acid, chemical structure.

RA is a vitamin A-derived metabolite critical for regulating immune functions. Vitamin A is metabolized to 11-cis-retinal after being ingested as carotenoids or retinyl esters, whereas all-trans-retinoic acid is the main intermediary of biological functions of vitamin A. All-trans-retinoic acid has the retinoic acid receptor (RAR) that heterodimerizes with retinoid X receptors [91]. Vitamin A deficiency increases the predisposition to many diseases, including diarrhea, measles, and respiratory infections. Hydrophobic molecules derived from vitamin A are called retinoids [105]. The primary molecules with critical functions are retinol (vitamin A) and retinoic acid, followed by the oxidized form of retinol, with the most common isomers being all trans-retinoic acid (AT-RA) and 9-cis retinoic acid (9-cis-RA) [114,115]. RA is known chemically as 3,7-dimethyl-9-(2,6,6-trimethyl-l-cyclohexen-l-yl)-2,4,6,8-nonatetaenoic acid (Figure 4) [116].

### Effect of Retinoic Acid on Gut Immunity

In the intestinal environment, DCs, effector T-cells, epithelial cells, macrophages, LB, ILC1, LTi, plasmatic cells, CD103 + DCs, Tregs, and Th17 [117] are cell types that can metabolize RA, which allows for the maintenance of the homeostatic environment. At the same time, RA can modulate the cellular differentiation and proliferation of these cell lines. These capabilities are attributed to its natural RA isomers 9-cis, 13-cis, and all-trans RA, depending on their affinity to the RA nuclear receptor [118], the receptors (RARs), and retinoid X receptors (RXRs) [119]. RA primarily improves DC maturation and function, including the antigen processing and presentation capacity [106,120,121,122], promotes the expression of CD1d in all APCs, and stimulates lipid presentation as well as the activation and proliferation of NK T-cells. The main effects of RA on immune gut cells are presented in Table 2.

The presence of RA is related to food tolerance, which is mainly attributed to the first exposure to food antigens, sensed by DCs, and recognized as dietary compounds.

By contrast, when the diet lacks RA, food recognition as a non-antigenic epitope can fail, and these antigens can trigger an inflammatory response, developing into inflammatory bowel disease, celiac disease, and many food allergies [123].

RA actively participates in the regulation of tissue and organ development [124] as well as cellular growth and is an essential component of cell-to-cell signaling during vertebrate organogenesis. RA also promotes homeostasis and cellular differentiation [125,126].

Recently, it has been shown that the stimulation of follicular DCs with bacterial products and RA can synergistically enhance the expression of the CXCL13 and BAFF chemokines as well as TGF-β secretion and function [127]. Interestingly, mucosal DCs can metabolize retinol into RA [128,129,130] and control the generation of migrating intestinal homing DC precursors (pre-UDCs) in bone marrow [38], which can differentiate into CCR9^+^ plasmacytoid and conventional DCs that preferentially develop into CD103^+^ DCs [131]. RA can promote FOXP3 regulatory T-cell differentiation [34] and IgA production [132]; it is also responsible for maintaining the balance between the immune response and tolerance, as well as the homeostasis of the intestinal barrier, which prevents the passage of antigens and toxins derived from pathogenic microorganisms [133].

Innate lymphoid cells (ILCs) are among the cell lineages recognized for their function in immunity homeostasis and the development of pathology and are the target in the treatment of many diseases, such as psoriasis. ILCs are divided into groups according to their function. The ILC3 lineage is responsible for gut homeostasis because of its high IL-22, IL-17, and GM-CSF production in the physiological state [134]. By contrast, the ILC1 group has been correlated with the chronic gut inflammatory process that could develop into inflammatory bowel disease through an IFN-γ-mediated inflammatory process [135].

RA modulates cellular differentiation and gut homing of ILC1 and ILC3 and mainly regulates the development and function of these cell lineages [136].

Furthermore, in a study, RA modulated the gut inflammation induced by dodecyl sulfate sodium (DSS) administration in a mouse model. The same results were observed in a model of *Citrobacter rodentium* infection [137]. RA was administered after bacterial infection; thereafter, γδ T-cells were induced to produce IL-22 by specific and direct binding to the IL-22 promoter, thus regulating the transcription of IL-22 mRNA. This mechanism was observed in an in vitro cell culture [138].

## 5. The Gut Microbiota and the Development of Metabolic Diseases

The gut microbiota has a plethora of regulatory functions such as satiety, lipid homeostasis, glucose regulation, the metabolism of non-digestible dietary substrates, residues, and cellular debris, vitamin production, and immunomodulation, in addition to its protective function against pathogenic microorganisms via antimicrobial secretion. Moreover, the microbiota produces metabolites and diverse substances that have anticarcinogenic and anti-inflammatory functions [139].

Endogenous and exogenous factors such as poor nutrition, stress, lack of physical activity, vitamin deficiencies, diseases, and antibiotics can induce microbiota dysbiosis. Dysregulation in the microbial environment for prolonged periods leads to the development of disturbances, mainly manifested as small intestinal bacterial overgrowth (SIBO). These disturbances can develop into metabolic syndrome, type 2 diabetes, obesity [140], and lipid disorders such as non-alcoholic fatty liver disease (NAFLD) [141].

It is known that the commensal pathogenic microbiota can disturb vitamin A metabolism. When the microbial environment is composed of beneficial microbiota, such as segmented filamentous bacteria (SFB), it can induce Th17 differentiation from naïve T-cells [142]. These bacteria affect the development and maturation of Th17 cells [143], which control mucosal fungus and bacterial infections by producing IL-22, IL-21, IL-17F, and IL-17A cytokines [144].

The balanced state of the microbial communities in the gut (i.e., eubiosis) depends on the age, genetic factors, nutrition, lifestyle, and health of the host. Conversely, dysbiosis involves an imbalanced gut microbiota community with characteristic toxin production, which increases epithelium permeability and promotes numerous immunological and hormonal changes. An HFD is one of the more common causes of gut dysbiosis because it promotes an imbalance in the growth ratios of Firmicutes/Bacteroides, the main microbial phyla in a healthy microbiota [145].

An HFD is responsible for a decrease in Th17 cells, which are crucial for the maintenance of the mucosal barrier structure and function [146]. IL-17 cytokine production by TH17 cells inhibits the pro-adipogenic transcription factor and modulates glucose metabolism in an obesity model induced by an HFD [147].

SFB promotes the release of serum amyloid A (SAA) proteins that induce IL-17 and IL-22. These cytokines, in turn, modulate the production of antimicrobial peptides by epithelial cells (IECs) in the intestinal lumen and IgA secretion [101].

Dysbiosis induced by a high-fat diet (HFD) facilitates the onset of metabolic diseases in reaction to the low-grade metabolic inflammation resulting from alterations in the intestinal microbiota [141]. These changes are associated with the development of obesity. Short-chain fatty acids (SCFAs) influence the populations of regulatory T-cells (Tregs), and these influences have the potential to regulate the induction of differentiation in Th1, Th2, and Th17, thereby preserving immune homeostasis [142].

A recent analysis reported that the total saturated fat intake is associated with the development of diseases derived from metabolic syndrome and high mortality risk, contrary to what happens with the consumption of medium- and odd-chain saturated fatty acids (SFAs), which is negatively associated with the mortality risk [148].

The composition of the intestinal host microbiota is related to the excessive intake of fats and refined carbohydrates, which affect its diversity. This promotes the growth of specific microbiota that produces LPS and has a high concentration in the plasma, which in turn modifies the immune response, resulting in low-intensity systemic inflammation that is associated with the development of obesity and metabolic disorders [149,150].

In mice with obesity induced by an HFD, only a lower bifidobacterium population was noted [151]. The presence of this bacterial genus is associated with glucose tolerance and considerably decreased inflammation [152], which indicates that these probiotics may have the potential to prevent the development of metabolic diseases. Accordingly, in many intestinal diseases, such as inflammatory bowel diseases, an unusual decrease in the number of bifidobacteria has been observed. Moreover, in the case of obesity, bifidobacteria dysbiosis precedes this development [153].

SCFAs, such as acetate, propionate, and butyrate, are metabolites of the saccharolytic fermentation of dietary fibers and resistant starch by commensal bacteria in the gut. Their proportion depends on the ratio of the beneficial gut microbiota, and they may change depending on the host’s age, diet, and diseases. These SCFAs play an important role as mediators in the communication between the gut microbiome and the immune system. They produce signals targeted to the immune cells by free fatty acid receptors (FFARs). In this manner, these SCFAs influence both the pro- and anti-inflammatory response through an immunomodulatory effect [154]. In older people, there is a relationship between the metabolite serum concentration and the microbiota population, with an imbalance in the proportion of Firmicutes/Bacteroidetes, wherein the Bacteroidetes proportion is higher. This corresponds to one of the typical characteristics of biological aging that can be favored by a considerable difference in the SCFA levels in feces affecting the metabolite production levels [155].

Harmful metabolites produced by the microbiota are associated with the secretion of MIP-2, TNF-α, and IFN-γ cytokines by macrophages. The production of these cytokines induces a decrease in the commensal microbiota. Nonetheless, RA oral administration can restore the Th17 population [156] and probiotic supplementation can modulate gut microbiota [52]. Moreover, LF induces the growth of many of the gut’s probiotic strains [157].

On the other hand, there is evidence that the gut microbiota can condition ILC activity. These ILCs induce pancreatic endocrine cells to produce β-defensin 14 (mBD14), a defensin that has a crucial function in pathogenic colonization, in addition to the prevention of the development of autoimmune diabetes, which was observed in a model employing non-obese diabetic rodents [158], where mBD14 was expressed in extremely low amounts because of a low and imbalanced gut microbiota population.

## 6. Effect of Retinoic Acid and Bovine Lactoferrin on the Gut Microbiota

We have already highlighted the importance of the intestinal microbiota. To emphasize this further, mice without microbiota were reported to show impaired immunological development characterized by an immature GALT, a small number of B- and T-lymphocytes, poor IgA secretion, and low levels of antimicrobial peptides and proteins [159,160]. However, such defects were corrected when the mucosa was colonized with commensal bacteria, demonstrating that such bacteria are necessary for the differentiation of Th17. These bacteria induce the IL-25 expression by epithelial cells, interfering with the production of IL-23 by MØs, which can suppress the development of Th17 [161]. Additionally, micronutrients and vitamins have been shown to have little effect on the microbiota. However, the signals from these nutrients can be amplified by intestinal epithelial cells (IECs) and other stromal cells that secrete mediators, in turn affecting the proliferation of luminal microbes [162,163]. These nutrients also stimulate IECs and mononuclear phagocytes to secrete B-cell-activating factor (BAFF) and proliferation-inducing ligand (APRIL) and TGF-β to promote IgA class-switching and assist in maintaining IgA+ antibody-secreting cells (ASCs). It has also been observed that RA facilitates the induction of IgA by commensal microorganisms, stimulating this process directly or indirectly via innate lymphoid cells (ILCs) [101,164].

RA has been observed to act as a cofactor in the stimulation of intestinal cells. Regarding the fluid intestinal levels of S-IgA in a model of vitamin A-deficient rats, a substantial decrease in secretory component (SC) production was reported [165].

DCs in the intestine produce RA by answering to the TGFβ signal, which is obtained in response to the microbiota sampling and environmental cues [166]. However, chronic contact between microbiota motifs and immune cells can result in the development of diabetes and obesity because of low-grade inflammation [167].

The RA production by CDs can be altered by microbial products and dietary changes.

RA enhances the production of immunoglobulin (Ig) by plasma cells in vitro [168], which can increase the secretion of IgA in all mucosal epithelia, amplifying immunoprotection. These increases are correlated, which suggests that RA can function as an important component for the maintenance of homeostasis within the epithelium of the intestinal mucosa, an effect that may be mediated by the induction of specific nuclear receptors for RA. In turn, this proves the essential contribution of RA in the diet to the maintenance of homeostasis.

Interestingly, it has also been observed that RA can act synergistically when combined with some proteins, such as TGF-β, with which isotype-switching is induced [169].

On the other hand, LF promotes the growth and expansion of the beneficial microbiota. This facilitates improvements in the permeability, growth, and maturation of the epithelial cell monolayer and nerve fibers, allows microbial antagonism, and regulates the pro- and anti-inflammatory response, therefore, promoting gut homeostasis [170].

IgA plays a major role in the control of the microbiota because it can react against some of its members, mainly Proteobacteria [171]. This group, known as the pathogenic microbiota, includes the major species related to intestinal and systemic inflammatory diseases. Thus, an increase in its population is related to dysbiosis [172]. IgA can bind to Proteobacteria, and a disruption in IgA production promotes dysbiosis and the increase in the Proteobacteria population. In metabolic syndromes such as diabetes, where conditions such as adiposity, obesity, inflammation, and insulin resistance are present, Proteobacteria are overexpressed and IgA production is diminished [171].

When RA is combined with LF, a synergistic effect that improves the IgA response occurs, with the increase restricted only to the IgA isotype [173].

The intrinsic vitamin A signal has been shown to exhibit a slight anti-infective capacity against pathogen invasion and can activate immune cells to eliminate the pathogen, removing it from the microbiota [174].

RA inhibits Th17 production by IL-6 and promotes anti-inflammatory Treg cell differentiation via TGF-β [156]. Th17 differentiation is mediated by retinoid-related orphan receptor-gamma t (RORγt), and cytokines such as IL-6, TGF-β, IL-1β, and IL-21 can induce its differentiation. IL-23 maintains this population. SFB are commensal bacteria necessary for Th17 development. The changes in homeostasis in the intestinal immune system, mainly in this cellular population, could trigger a metabolic disease such as type 2 diabetes [28].

Diets with a high amount of dietary fat contribute to microbiota dysbiosis and, consequently, to the decrease in the number of Th17 cells based on the low capacity of APCs to stimulate Th17 differentiation. Likewise, there is a decrease in neutrophil activation, which was verified by the expression of myeloperoxidase by neutrophils during a respiratory burst [175]. All these downregulated processes promote the establishment of pathogenic microbiota and contribute to the generation of an exacerbated inflammatory process, driving the development of colitis or metabolic diseases such as type 2 diabetes mellitus [28,176]. In dysbiosis associated with HFDs and inducing intestinal inflammation, high concentrations of harmful metabolites such as LPS are generated because of the production of monosaccharides and SCFAs from indigestible polysaccharides and their storage as fat [177]. These metabolites are recognized by PRRs, mainly by TLRs, and induce a proinflammatory response with the production of cytokines, resulting in insulin resistance [151].

In summary, when there is a persistent interaction between the intestines and an HFD, the intestines exhibit a chronic inflammatory response, which is correlated with obesity and insulin resistance development [178] and can promote the proliferation of unhealthy microbiota. The colonization by these pathogenic bacteria promotes the systemic accumulation of toxic products derived from these microorganisms, such as LPS and saturated fatty acids, increasing the inflammatory process due to their interaction with the TLR.

On the other hand, LF can hamper body weight gain and the adipose index and prevent hypercholesterolemia and hyperglycemia in addition to its nutritional value [179]. In the case of a downregulated intestinal activity, LF can regulate dysbiosis by the modulation of the inflammatory response.

## 7. Conclusions and Perspectives

The microbiota plays an important role in regulating both innate and adaptive immune responses. When the homeostasis of the microbiota, environmental cues, and its host is dysregulated, inflammatory processes occur, and an imbalanced state is generated. If these factors persist for a long period, they might trigger the development of metabolic diseases that are mainly related to processes such as the lack of or decrease in antigenic tolerance toward the microbiota and the consequent inflammatory and inefficient regulatory response by Th17 cells and Tregs. Interestingly, both LF and RA can promote the maturation of the intestinal immune system and, consequently, reduce inflammatory processes, whereas LF can act positively on the microbiota population. This favors the establishment and growth of microbes beneficial to the host, such as Firmicutes, Bacteroides, and bifidobacteria.

Probiotics, with their positive impact on gut health, have been increasingly recognized for their beneficial effects in reducing the risk of developing metabolic diseases. A balanced and diverse gut microbiota, promoted by the regular intake of probiotics, plays a pivotal role in metabolic health. Probiotics contribute to maintaining a healthy balance in the gut flora, influencing factors such as inflammation and insulin sensitivity. By fostering a favorable microbial environment, probiotics can help regulate metabolic processes, including glucose metabolism and lipid profiles. Probiotic supplementation may contribute to improved weight management, reduction of abdominal fat, and mitigation of insulin resistance. These findings suggest that incorporating probiotics into one’s diet may serve as a proactive and natural approach to support metabolic health and potentially reduce the risk of metabolic diseases, such as type 2 diabetes and cardiovascular disorders. Nevertheless, individual responses to probiotics may vary, and it is advisable to consult with healthcare professionals for personalized recommendations.

With this information, we wish to highlight the importance of the microbiota and the intestinal environment in the regulation of many (if not all) immune processes of the intestine, and vice versa, in terms of both health and disease. Still pending is the demonstration of the synergistic regulatory effect of LF and RA in both in vivo and in vitro models of dysbiosis associated with metabolic diseases. Thus, we want to find out whether, together with the establishment of beneficial and new microbiota, these chronic inflammatory processes can modify the balanced profile of Th1 and Th2, as happens in newborns when exposed to proteins in breast milk (mainly colostrum). This condition allows the proper colonization of the intestinal microbiota, combined with proper nutrition.

In conclusion, the proper use and application of the synergistic effect between bovine lactoferrin and retinoic acid appear promising. The collaborative action of these compounds can potentially improve therapeutic outcomes in areas such as immune modulation, cellular integrity, and other physiological functions. Further exploration of their combined effects in various contexts, supported by the information discussed in this article, may reveal new opportunities for therapeutic interventions and health promotion. As we deepen our understanding of the beneficial interaction that may result between bovine lactoferrin and retinoic acid, this synergy emerges as a compelling avenue to optimize health benefits and address a spectrum of health-related challenges, such as preventing the development of diseases such as obesity, diabetes mellitus, and other metabolic disorders.

## Figures and Tables

**Table 1:** LF and RA biological characteristics and main synergistic effects.

Protein	Distribution	Related Functions	Synergic Effects (LF/RA)
Lactoferrin (LF)	Colostrum, human milk, and mucosa	Activation and regulation of the immune system	IgA class-switching Increasing CCR9 and α4β7
Retinoic acid (RA)			

**Table 2:** Multiple effects of RA and LF on immune gut cells.

Molecule	Activity	Administration Time	Administration Route	Effect on Target	Activation Target	In Vitro/In Vivo Studies	Reference
Lactoferrin	Immune gut homeostasis	At birth	Breastfeeding	Bifidobacteria	T-cell	In vivo	[91]
	Bacteroides gut colonization	Newborn infant	Breastfeeding	IgA binding	IgA	In vivo	[92]
	Growth promoter	Mice	Diet	Growth promotion	Lactobacillus and Bifidobacteria	In vivo	[91]
	DC’s	Any stage of life	Diet	Immunity homeostasis	DC’s	In vitro	[93]
	Anti-inflammatory bacteria population	Any stage of life	Diet	Restoring TLR level expression	TLR receptor expression	In vivo	[99]
Retinoic acid	Maintenance of the homeostatic immune environment	Cell culture	On culture	Maduration, differentiation, and prolifferation modulation	DC’s T-cell Epithelial cells MQ’s B lynphocites IlC1 LTi Plasmatic cells CD103 + DC	In vitro	[100]
	DC’s function	Cell culture	On culture	Improve maturation and function, antigen processing, and presentation capacity	CD1d DC’s expression	In vitro	[101,102,103,104]
	DC’s molecules expression	Cell culture	On culture	Improve CXCL3 expression	CXCL13, BAFF, TGF-β	In vitro	[105]
	ILC1 and ILC3 modulation	Cell culture	On culture	Cellular diferentiation, gut homing, development, and function modulation	IL-22, IL-17 and GM-CSF production	In vitro	[106]
	Food tolerance	Cell culture	On culture		DCs and gut-tropic FOXP3 + regulatory T-cells	In vitro	[107]
